# Telecare Service Use in Northern Ireland: Exploratory Retrospective Cohort Study

**DOI:** 10.2196/22899

**Published:** 2022-05-31

**Authors:** Hala Al-Obaidi, Feras Jirjees, Sayer Al-Azzam, Verity Faith, Mike Clarke, Evie Gardner, Ashley Agus, James McElnay

**Affiliations:** 1 Department of Clinical Sciences College of Pharmacy and Health Sciences Ajman University Ajman United Arab Emirates; 2 School of Pharmacy Queen's University Belfast Belfast United Kingdom; 3 Department of Pharmacy Practice and Pharmacotherapeutics College of Pharmacy University of Sharjah Sharjah United Arab Emirates; 4 Faculty of Pharmacy Jordan University of Science and Technology Irbid Jordan; 5 Northern Ireland Clinical Trials Unit Health and Social Care in Northern Ireland Belfast United Kingdom

**Keywords:** telecare, Northern Ireland, assistive technology, elderly people, healthcare use

## Abstract

**Background:**

Telecare is a health service that involves the home installation of a number of information technology support systems for individuals with complex needs, such as people with reduced mobility or disabilities and the elderly. It involves the use of sensors in patients’ homes to detect events, such as smoke in the kitchen, a front door left open, or a patient fall. In Northern Ireland (NI), outputs from these sensors are monitored remotely by the telecare team, who can provide assistance as required by telephone or through the emergency services. The facilitation of such rapid responses has the aim of promoting early intervention and therefore maintaining patient well-being.

**Objective:**

The aims of this study were to construct a descriptive summary of the telecare program in NI and evaluate hospital-based service use by telecare patients before and after the installation of telecare equipment.

**Methods:**

An exploratory retrospective cohort study was conducted involving more than 2000 patients. Data analysis included the evaluation of health care use before and after the telecare service was initiated for individual participants. Individuals with data for a minimum of 6 months before and after the installation of the telecare service were included in this analysis.

**Results:**

A total of 2387 patients were enrolled in the telecare service during the observation period (February 26, 2010-February 22, 2016). The mean age was 78 years (median 81 years). More women (1623/2387, 68%) were enrolled in the service. Falls detectors were the most commonly deployed detectors in the study cohort (824/1883, 43.8% of cases). The average number of communications (calls and/or alarms) between participants and the coordinating center was the highest for patients aged ≥85 years (mean 86 calls per year). These contacts were similarly distributed by gender. The mortality rate over the study period was higher in men than women (98/770, 14.4% in men compared to 107/1617, 6.6% in women). The number of nonelective hospital admissions, emergency room visits, and outpatient clinic visits and the length of hospital stays per year were significantly higher (*P*<.001) after the installation of the telecare equipment than during the period before installation.

**Conclusions:**

Despite the likely benefits of the telecare service in providing peace of mind for patients and their relatives, hospital-based health care use significantly increased after enrollment in the service. This likely reflects the increasing health care needs over time in an aging population.

## Introduction

It has been claimed that home-based telecare, particularly for the elderly, reduces the need for community care, prevents unnecessary hospital admissions, and delays or prevents admission into residential or nursing home care [1-6]. Telecare Northern Ireland is a service which provides a range of information technology support services to assist mainly elderly people who live independently in their own homes. It typically involves the use of sensors placed in patients’ homes to allow for the detection of critical events, such as smoke in the kitchen, a tap left running, a front door left open, or a patient fall [7-9]. The sensors allow for the transmission of alerts to a central coordinating center, from which staff respond as appropriate. Telecare can be used by a full spectrum of patients but is mainly used by elderly people who live alone in their own homes [4,5,9].

In 2008, the Minister for Health, Social Services, and Public Safety for Northern Ireland (NI) announced £1.5 million (US $1.97 million) funding for pilot projects to promote the development of telehealth [3]. A telecare program was introduced as part of this initiative, under the umbrella of a more extensive Telemonitoring NI initiative. At the time the cohort of users was established, there were approximately 1.7 million people in the United Kingdom (UK) using telecare services [10].

Telecare programs in NI typically involve the deployment of different equipment and/or sensors, depending on the perceived benefits to the patient. Although telecare has the potential to play an important role in enhancing the ability of elderly people to manage their activities of daily living and, if required, avail of rapid response services, there is often a misunderstanding regarding the role of the service. It should be considered an aid to improve elderly patients’ independence and quality of life and not a solution to their growing need for general health care and hospital-based care [3-5,9].

A private company (TF3) won the contract to provide telecare services in NI. The UK National Health Service (NHS) operates in NI, and the telecare service, as is the case with other health care services, is free of charge to patients. Patients were enrolled in the program by their clinical team. The range of sensors and components deployed in the NI program were as follows: a pendant that the patient can activate if he or she experiences a fall, an emergency alarm button, an extreme temperature sensor, a bed or chair occupancy sensor, a home safety package (consisting of a pressure mat, bogus caller button, epilepsy sensor, and property exit sensor), a fumes detector, a flood detector, and an immobility sensor. The combination of components used with each patient was adjusted according to individual patient needs. The equipment was installed and maintained in patients’ homes by TF3 and all patients across NI were connected to a call center that dealt with alarms and calls from patients receiving the service.

The aims of this exploratory study were to construct a descriptive summary of the use of the telecare program in NI and evaluate patient use of hospital-based services before and after the introduction of the telecare service.

The objectives were as follows:

- Using patient administrative data collected by the provider of telecare services in NI (TF3) as part of service provision, together with health care use data sets held at the Business Services Organisation (BSO) in NI, develop a descriptive summary of the patients enrolled in the telecare service from 2010-2016.
- Using data held by TF3 and Health and Social Care in NI (HSC), compare hospital-based service use before and after telecare service initiation in patients’ homes.

## Methods

### Ethics Approval

Ethical approval was obtained from the National Research Ethics Service Committee (Research Ethics Committees 15/SW/0015, SET/14/68, WT/14/37; Integrated Research Application System project ID: 167795). Governance approvals and data access agreements were approved by the HSC Trusts.

### Data Access and Confidentiality

Access to the health care data sets of individual NHS patients in NI is only made available to researchers in anonymized form via a confidential data repository (ie, the Honest Broker Service [HBS], established by the BSO in NI. The HBS provides a “safe haven” in which data can be accessed and analyzed within a confidential secure environment).

Patient-level data supplied by TF3 and the HSC Trusts were anonymized by the HBS and made available to the research team. To ensure confidentiality, identifiable data are not accessible to researchers and results from analyses undergo scrutiny before being released.

### Data Acquisition and Inclusion in Master Data Set

Health care use data (ie, nonelective hospital admissions, periods of hospital stay, outpatient clinic visits, and emergency room [ER] visits) were obtained for all enrolled patients.

Individual patient data sets were retrieved using Health and Social Care numbers (HCNs) for the period before and after the installation date. If a patient died after installation, the date of death was inputted as the endpoint for that individual. The HCN is a unique identifier for all patients registered to receive NHS services in NI and was crucial for data linkage. The date of telecare equipment installation was used as the cut-off point to demarcate preservice and postpatientservice use. Following clearance by the data guardians at the 5 HSC Trusts, TF3 provided data sets on telecare usage to the HBS for linkage and access by the research team. Patients who had data relating to a minimum of 6 months before and after the initiation of the telecare service were included in the health care use aspect of the study.

### Data Analysis

The data were analyzed in the HBS using SPSS (version 22; IBM Corp). Descriptive data analyses on patient demographic characteristics (eg, age and gender), number of calls (communications between the patient and coordinating center), telecare equipment components installed, and mortality rates were performed. Differences in the continuous variables relating to health care use before and after telecare installation were tested for significance using the paired *t* test.

## Results

### Demographic Data

Data for a total of 2387 patients enrolled in the telecare service in NI indicated that more female patients than male patients received the telecare service (n=1623, 68% female patients compared to n=764, 32% male patients). The mean age of participants was 78 (SD 12) years; 1716 (1716/2387, 72%) individuals in the study population were 75 years or older. Only 295 (295/2387, 12%) were under 65 years of age.

### Contact Calls by Age Group and Gender

Out of the 2387 patients enrolled to receive the telecare service, 2330 patients had records of contact with the coordinating center (eg, in a fall alarm event, both the incoming alarm and outgoing call were recorded and counted as 2 calls). There were between 1 and 7183 calls per patient per year, with a mean of 64.7 and a median of 33 calls per year.

The highest average number of annual patient contact calls was in the ≥85 years age group, with an average of 86 calls per year. This decreased to 59 calls per year in the 75-84 years age group and 54 calls per year in the 65-74 years age group. In addition, the lowest mean number of calls (47 calls per year) was in the ≤64 years age group. Finally, the average number of calls was very similar for female (65.6 calls per year) and male patients (62.8 calls per year).

### Mortality of the Enrolled Patients

A total of 205 (205/2387, 8.6%) of patients died during the observation period. As expected, the mortality rate was the highest in patients who were ≥85 years when they were first enrolled. Mortality during the observation period was more than 2 times higher in male participants (98/770, 12.7%) compared with female participants (107/1617, 6.6%).

### Installation Frequency of Telecare Equipment Components

**Figure 1:**
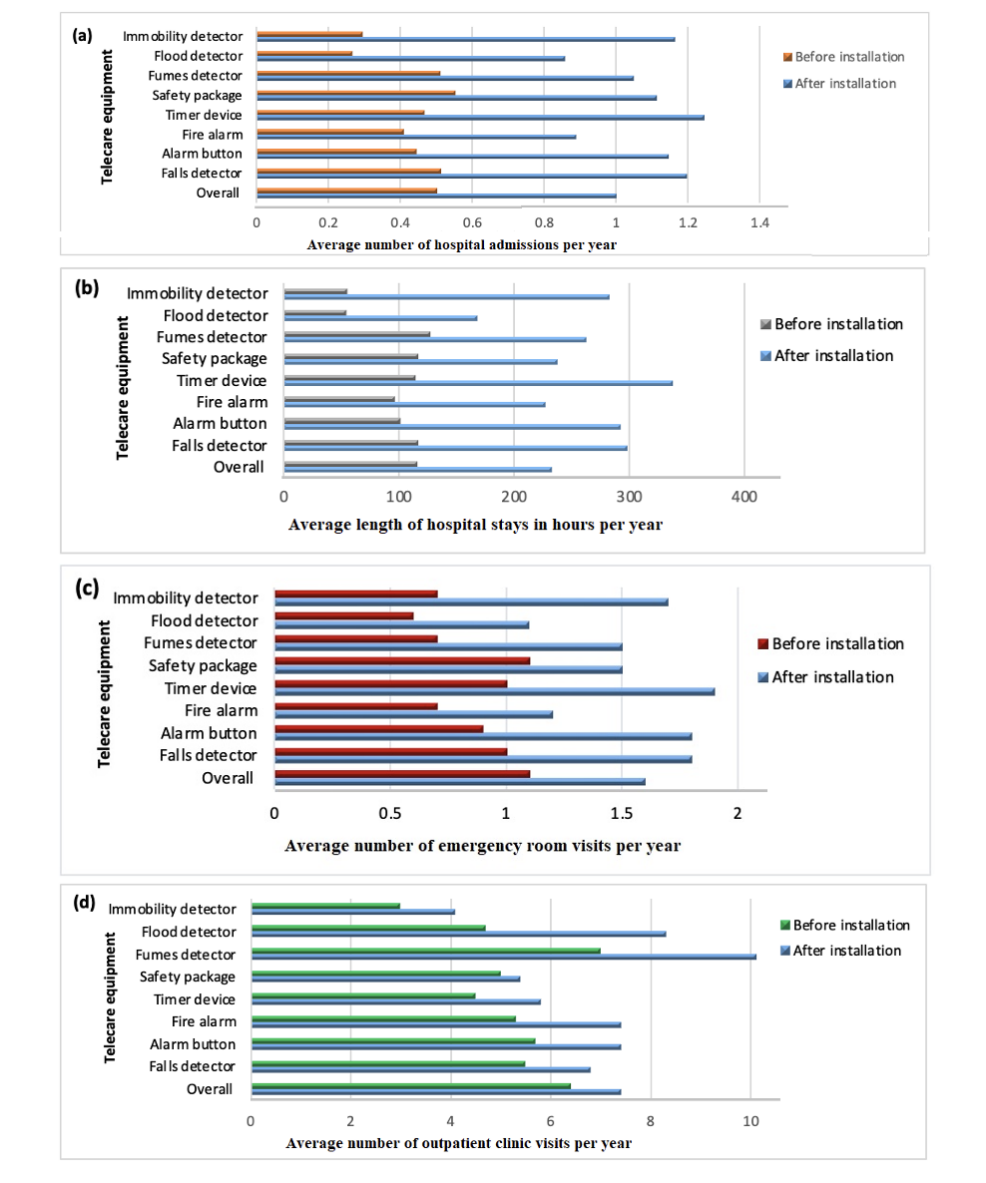
The mean (a) number of nonelective hospital admissions per year, (b) length of hospital stays in hours per year, (c) emergency room visits per year, and (d) outpatient clinic visits per year for patients with differing telecare equipment components pre and post installation (n=1883).

Out of a total population of 2387 patients, 1883 patients had data available on the individual telecare equipment components installed in their homes. Data showed that almost all (1867/1883, 99.2%) of the patients had a call advisor or home unit installed. This equipment provides an alternative to a landline telephone and allows the patient to contact the call center. A total of 824 patients had a fall detector installed (a pendant that a patient can activate if he or she has a fall). The remaining telecare equipment components or detectors (shown in Figure 1) were less commonly installed. This includes the alarm (a button that a patient can activate in case of any emergency; 441 cases), fire alarm (an extreme temperature sensor; 276 cases), timer (a bed or chair occupancy sensor that has a timer device that can be set according to each individual’s routine and is placed under their mattress or chair cushion; 181 cases), safety package (consisting of a pressure mat, bogus caller button, epilepsy sensor, and property exit sensor; 96 cases), fumes detector (detects dangerous levels of carbon monoxide; 82 cases), flood detector (detects if water has overflowed onto the patient’s floor; 38 cases), and immobility sensor (detects lack of movement within the patient’s home, which suggests that patient has collapsed; 37 cases).

### Health Care Use Pre and Post Installation of the Telecare Components

The health care use parameters increased significantly after the installation of the telecare equipment. For example, the average number of nonelective hospital admissions per year increased from 0.5 (SD 0.6) to 1.0 (SD 1.5; *P*<.001), the average length of hospital stays increased from 115.3 (SD 190.6) to 232.2 (SD 485.2) hours per year (*P*=.006), the average number of ER visits increased from 1.1 (SD 1.7) to 1.6 (SD 2.5) visits per year (*P*<.001), and the average number of outpatient visits increased from 6.4 (SD 8.4) to 7.4 (SD 8.7) visits per year (*P*<.001). The results presented in Figure 1 illustrate these data with reference to the telecare equipment installed in patients’ homes.

## Discussion

### Study Focus

This study had a narrow focus (ie, to construct a descriptive summary of patients enrolled in and the use of the telecare program in NI and to evaluate hospital-based service use by patients before and after installation of the telecare equipment). In interpreting the results, one must consider that there is often tension within health and social care provision regarding the value versus the cost of various services since different services have different impacts on clinical, humanistic, and economic outcomes.

### Patient Age and Gender

The highest proportions of telecare NI patients were in the elderly age groups, with only 12% (295/2387) of participants under the age of 65 years. A total of 12% of the total UK population of >66 million people are aged ≥65 years [11]. Aging statistics in NI, which had a total population of 1.9 million in February 2021, show that the total number of people aged ≥65 years has increased from 13% (mid-1994) to 16.6% (mid-2019) [12]. This age group is projected to grow in all Great Britain (GB) regions by mid-2028 [13] (ie, there is a growing elderly population who may avail of telecare services).

The patients who enrolled for this telecare service were predominantly aged 75 years and above (1716/2387, 72% of the study population). The profile of participant age groups in this study is similar to the patients who enrolled for telecare services in England in the telehealth whole system demonstrator (WSD) project, in which approximately 60% of participants (intervention group) were aged ≥75 years [7]. The mean age of the patients enrolled for the telecare service in NI was 78 (SD 12) years, while it was 75 (SD 14) years for patients in the telecare arm of the WSD project [7].

All patients enrolled in this study were still able to live in their own homes or sheltered accommodation and were deemed able to take care of themselves with the aid of telecare equipment. In GB, approximately 60% of women aged ≥75 years live alone in their own homes, compared with 36% of men of the same age [14]. This disparity in the female-to-male ratio was evident in the uptake of telecare services in NI (1218:499 for patients aged ≥75 years).

Overall data on gender showed that 68% (n=1623) of the 2387 patients enrolled in the telecare service in NI were female. This is similar to a telecare service in Scotland, where 62% of a cohort of 7487 patients was comprised of female patients [5]. A similar male-to-female ratio was reported in the WSD project in England, where 67.5% of patients in both the control (n=1236) and intervention (telecare arm) groups (n=1190) were female [7].

### Patient Contact Calls

Despite the high level of activity in this study (eg, an average of 86 contacts annually between the telecare center and patients >85 years), markers of the need for hospital-based care increased over time among patients enrolled in the program.

Research by others has indicated more nuanced outcomes; for example, a systematic review [15] on the benefits of home telecare services for elderly patients involving 21 randomized trials and 12 observational studies found that regular calls between health care providers and patients reduced or delayed hospital admissions and improved discharge rates in elderly people with long-term conditions, leading to cost savings. The observational studies in the systematic review also indicated that supplementing the type of telecare service delivered in NI with daily follow-up telephone calls from nurses may further reduce costs by delaying hospital admissions and lowering the number of readmissions in elderly patients with heart disease, diabetes, and chronic obstructive pulmonary disease. However, the review found insufﬁcient rigorous evidence about the effects of safety and security alert systems, such as fall detectors and community alarms, on either individual or system outcomes [15]. A more recent study conducted in England found that the number of requests for ambulances as a consequence of falls was reduced by the rapid response of a telecare call center [6].

### Mortality

In NI, mortality rates have decreased in recent years across all age groups, but the mortality rates in men remain higher than in women. It has also been noted, however, that although women live longer, they often live the extra years in poor health [14]. These data help explain the greater use of the telecare service by women and their lower mortality in this study.

### Telecare Equipment Installation and Health Care Use

In NI, as in other locations, a wide assortment of sensors and devices were used according to the perceived needs of clients [9,16-18]. After the advisor call unit, the most frequently installed was fall detection equipment (824/1883, 44%). Falls are particularly problematic in an aging population and can have serious consequences, including bone (especially hip) fractures [6,19].

Although telecare is increasingly being used across GB, there has been little definitive work on its impact on health outcomes [20,21]. The variety of equipment components makes the delivery of randomized trials complex and difficult to perform [3]. The range and combinations of telecare equipment components used in different regions and countries, coupled with differing health and social care delivery models, also make it difficult to compare data from different centers.

An increase in health care use over time is to be expected in this study population because the majority (1716/2387, 72%) who enrolled were aged 75 years or older. Because a control group of people with similar characteristics who did not receive the telecare services was not available, the impact of telecare could not be evaluated; however, a doubling of the mean number of hospitalizations (0.5 to 1.0; *P*<.001) was disappointing and clearly highlights the impact of aging on health and well-being.

These findings can be considered alongside a report which summarized details of the Scottish Telecare Development Program [22]. This telecare provision was implemented at the time of hospital discharge over a 1-year period (2007-2008). As in this NI study, there was no control group. A total of 7902 patients were provided with the telecare service (85% aged ≥65 years). It was estimated (by the 18 telecare service providers involved) that more than 500 delayed discharges were avoided by the use of telecare, saving an estimated >5000 bed days. It was also estimated that more than 1200 emergency admissions were avoided, saving an estimated 13,000 bed days [22].

The demographics of the NI telecare recipient population were similar to those of the participant population in the study in Scotland. Since both regions operate under the UK NHS system, it is likely that benefit was accrued from the telecare service in NI despite the increased use of hospital-based services post installation. Peace of mind (through feeling safe and secure) achieved by both patients and their families, as demonstrated by other researchers [6,9,22-24], was likely to have been achieved, but this benefit could not be assessed using the NI data.

### Conclusions

Despite the likely benefit of the telecare service, including peace of mind for patients and their relatives [23,24], hospital-based health care use significantly increased after enrollment in the service. This may simply reflect the increasing health care needs due to health deterioration over time within an aging population; with no control data available, it was not possible to quantify the impact of the telecare service.

This quantification would require a new prospective study with a control group and, therefore, a randomized controlled trial is recommended to fully evaluate the potential of telecare services to improve clinical, humanistic, and economic outcomes across NI. This should be supplemented by a substantive qualitative aspect to the research, including interviews with both patients and their next of kin and the development of a number of case studies involving patients who engaged with the telecare service.

## Abbreviations

BSO: Business Services Organisation
ER: emergency room
GB: Great Britain
HBS: Honest Broker Service
HCN: Health and Social Care number
HSC: Health and Social Care in Northern Ireland
NHS: National Health Service
NI: Northern Ireland
UK: United Kingdom
WSD: whole system demonstrator
